# From neglect to necessity: The case for routine work-up of nontoxigenic *Corynebacterium diphtheriae* in clinical microbiology

**DOI:** 10.4102/ajlm.v14i1.2802

**Published:** 2025-09-17

**Authors:** Kgaogelo R. Masemola

**Affiliations:** 1 Department of Medical Microbiology, National Health Laboratory Service, Durban, South Africa; 2 School of Laboratory Medicine, College of Health Sciences, University of KwaZulu-Natal, Durban, South Africa; 3 Department of Medical Microbiology, Prince Mshiyeni Memorial Hospital, Durban, South Africa

## Introduction

Toxigenic *Corynebacterium diphtheriae* is well known for causing classical respiratory diphtheria, a life-threatening disease controlled by widespread toxoid vaccination.^1,2^ Nontoxigenic *C. diphtheriae* strains (those lacking diphtheria toxin production), on the other hand, have received less attention.^1,3^ However, nontoxigenic *C. diphtheriae* infections are being increasingly reported globally in recent times.^4^ For years, clinical laboratories often dismissed corynebacteria from non-respiratory sites as ‘diphtheroids’ (skin or mucosal commensals/contaminants), and did not identify them to species level.^5^ This practice has led to under-recognition of infections by nontoxigenic *C. diphtheriae*.^1^ With improvements in identification methods and accumulated case reports, nontoxigenic *C. diphtheriae* is now understood as an emerging pathogen capable of causing invasive disease, even in immunised populations.^5,6^ The aim of this article is to underscore why ‘diphtheroids’ should no longer be routinely overlooked as insignificant commensals dismissed as contaminants, and to encourage laboratories and clinicians to recognise and appropriately manage nontoxigenic *C. diphtheriae* infections.^5^

### Global perspective

Nontoxigenic *C. diphtheriae* infections have been increasingly reported since the late 20th century.^1,4^ These infections often occur in socioeconomically marginalised groups and settings (Table 1) where the organism can colonise damaged skin.^7^ In the 1980s and 1990s, outbreaks and case clusters were documented in various regions.^4,8^ By the mid-1990s, experts were already warning of the resurgence of *C. diphtheriae* and the rise of nontoxigenic *C. diphtheriae* in hospital settings.^4^ Studies and surveillance reports have indicated that nontoxigenic *C. diphtheriae* infections are rising, often linked to intravenous drug use, homelessness, and skin infections.^7,9^

**TABLE 1:** Selected reported outbreaks and case series of nontoxigenic *Corynebacterium diphtheriae*.

Location	Period	Key population and/or risk factors	Cases and manifestations	References
United States	1972–1982	Homeless with a history of alcohol dependence	Three outbreaks of cutaneous and pharyngeal diphtheria in marginalised adults; second and third outbreaks were nontoxigenic	^10^
Switzerland	1994	Injection drug users (needle-sharing)	Outbreak of invasive infections by one clone of nontoxigenic *Corynebacterium diphtheriae*	^11^
France	1987–1993	Homeless with a history of alcohol dependence	Thirty four cases of bacteraemia (half of these patients developed endocarditis); *36% mortality rate*	^12^
Australia	1990–1991	Pre-existing cardiac abnormalities, and intravenous drug user	Seven cases of endocarditis; *1 death*	^13^
Canada	2000–2005	Economically marginalised urban community with high rates of substance use and unstable housing	Seven cases of *Corynebacterium diphtheriae* bacteremia (6 nontoxigenic) with endocarditis in one case; predominant clone identified; *2 deaths*	^8^
Poland	2004–2012	Economically marginalised with a history of alcohol dependence	Twenty five reported cases (18 invasive); single highly pathogenic clone (sequence type 8) identified in 24 isolates over 9 years	^14^
Germany	2016–2017	Homeless, intravenous drug users in urban centres (Berlin, Hamburg)	Marked increase in nontoxigenic isolates; multiple clusters of sequence type 8 strains causing wound infections and some bacteremia	^7^
United States	2018–2023	Homeless, illicit drug use	One hundred and sixty six patients; ~78% isolates from wound infections (often polymicrobial), 12% from blood; 6 cases of endocarditis; *8% overall mortality*	^15^
South Africa	2021	Rural community, mixed risk profile	Cluster of 5 endocarditis cases over 2 months; all due to one novel strain of nontoxigenic *Corynebacterium diphtheriae*; aggressive course with *4 deaths*	^1^

*Source*: Please see full reference list of this article, Masemola KR. From neglect to necessity: The case for routine work-up of nontoxigenic Corynebacterium diphtheriae in clinical microbiology. Afr J Lab Med. 2025;14(1), a2802. https://doi.org/10.4102/ajlm.v14i1.2802, for more information.

### African perspective

African cases of nontoxigenic *C. diphtheriae* infections are less well documented, likely because of underreporting.^1,2^ Despite this, there is growing recognition that these infections also occur in Africa and can be severe.^1^ South Africa has reported sporadic invasive cases over the years such as an outbreak of five fatal nontoxigenic endocarditis cases in 2021.^1^ Unlike Europe and North America, where nontoxigenic *C. diphtheriae* surveillance is more documented, African epidemiological data remain sparse. The true burden of disease is therefore unknown.

## Clinical manifestations and disease spectrum

Nontoxigenic *C. diphtheriae* infections can present in a variety of ways. Cutaneous diphtheria manifests as chronic, non-healing ulcers, or sores, which often serve as reservoirs for transmission.^10,14^ Invasive bloodstream infection and endocarditis are the most severe form of nontoxigenic *C. diphtheriae* disease, with a high mortality rate.^1,8^ Septic arthritis and osteomyelitis are less common, but are serious infections which often occur in the context of trauma or injection drug use.^16^ Nontoxigenic strains do not cause classic respiratory diphtheria, but they can colonise the throat and cause sore throat or tonsillitis.^6^ Other rare manifestations of nontoxigenic *C. diphtheriae* are pneumonia, urinary tract infections (especially in catheterised patients), and other localised infections.^3,15^

## Patient outcomes

The mortality associated with nontoxigenic *C. diphtheriae* infections varies depending on the type and severity of the infection, as well as patient-specific factors.^3^ In a study in Seattle, 9 (20%) of 44 patients with *C. diphtheriae* detections had death documented in the medical records.^3^ Nontoxigenic *C. diphtheriae* infective endocarditis has a mortality rate ranging from 35% to 66%.^3^ In one cluster of endocarditis cases, the 30-day mortality rate was 80%.^1^ Three of the four endocarditis patients in the Seattle study died from endocarditis-related complications within 1 week of presentation.^3^

## Laboratory diagnosis of *Corynebacterium diphtheriae*

The laboratory work-up for *C. diphtheriae* is typically undertaken when there is a strong clinical suspicion of diphtheria or in the context of an outbreak. For example, during the 2015 KwaZulu-Natal diphtheria outbreak, laboratories in South Africa were advised to incorporate selective media for the isolation of *C. diphtheriae* when processing throat swabs.^2^ However, in the absence of these defined scenarios, coryneform bacteria are seldom investigated beyond being classified as ‘diphtheroids’ or ‘Coryneform bacteria’, with many isolates dismissed as contaminants.^5^

Incidental identification of *C. diphtheriae* has increased with the widespread adoption of advanced technologies such as Matrix-Assisted Laser Desorption/Ionization Time-of-Flight Mass Spectrometry (MALDI-TOF MS), which provides rapid species-level identification.^3^ In cases where further work-up is pursued, laboratory protocols typically involve:

- Gram staining to identify pleomorphic, Gram-positive bacilli with a club-shaped morphology, often arranged in angular ‘Chinese character’ formations.^8,9,13^
- Standard culture media, including 5% sheep’s blood agar, chocolate agar, and MacConkey agar, are inoculated, with additional biochemical testing performed on suspect colonies.^8,13^ Some laboratories opt to subculture onto selective media such as Cystine tellurite blood agar or Tinsdale medium, where *C. diphtheriae* produces characteristic black colonies with brown halos.^17^
- Genus and species identification is then confirmed using commercial systems such as Analytical Profile Index assays or MALDI-TOF MS, with isolates in some countries being referred to national reference laboratories for confirmation and toxin determination.^17^
- The modified Elek test is used for phenotypic expression, while polymerase chain reaction detects the *tox* gene.^17^

Traditionally, antimicrobial susceptibility testing is not routinely performed. The European Committee on Antimicrobial Susceptibility Testing body has published guidelines for susceptibility testing of *C. diphtheria* and *C. ulcerans*.^18^

## Barriers and practical implications

While this article is framed as an advocacy argument for the routine species-level identification of Corynebacteria, the author fully acknowledges that there are practical and cost considerations to be kept in mind. These include:

- Lack of access to diagnostic advances such as MALDI-TOF MS, Analytical Profile Index Coryne kits and molecular diagnostics
- Lack of microbiology reference laboratory support
- Cost of selective culture media and confirmation testing
- Many so-called diphtheroids may actually be unrelated skin or environmental bacteria, not true Corynebacteria
- Increased workload in clinical laboratories because of routine work-up of Coryneform bacteria
- Difficulty in ascertaining the clinical significance of Corynebacteria in polymicrobial cultures
- Lack of clinical information accompanying culture requests.

These practical considerations are not insignificant, and any potential change must account for this. It is within this context that a shift in approach is being suggested.

## Paradigm shift in the laboratory approach to *Corynebacterium diphtheriae*

A transition is required in the approach to coryneform bacteria within clinical microbiology laboratories. This shift must also include improved communication between clinicians and laboratory staff. This will ensure that relevant clinical context accompanies specimen submissions and therefore guide appropriate interpretation of findings. The identification of *C. diphtheriae* can no longer be relegated to incidental findings.^5^ If this remains the case, its epidemiology will remain poorly understood, and the true burden of disease will continue to be underestimated.

To address this emerging threat, the following steps are critical:

1. Microbiology laboratories should have a heightened index of suspicion for nontoxigenic *C. diphtheriae*.
2. Public health agencies should integrate nontoxigenic *C. diphtheriae* into antimicrobial resistance surveillance programmes to track trends in antimicrobial resistance and virulence.
3. Investment in cost-effective diagnostic tools for resource-limited settings is essential for improving detection.

The longstanding practice of routinely dismissing coryneform species-level identification must be reconsidered. To ensure accurate identification and reporting, laboratories should consider diagnostic workflows that incorporate selective media, species confirmation using MALDI-TOF MS or Analytical Profile Index, and toxin determination through reference laboratory testing (Table 2).

**TABLE 2:** Recommendations for the identification, confirmation, and surveillance of *Corynebacterium diphtheriae*.

1. Specimen processing	Ensure skin and wound specimens are inoculated onto selective media alongside standard microbiological culture media to enhance detection.
2. Microscopic examination	Perform Gram staining on suspect colonies to confirm if Gram-positive bacilli with palisade formations or characteristic ‘Chinese character’ are present.
3. Culture and identification	Confirm species identity using commercial systems such as MALDI-TOF MS or API.
4. Reference laboratory confirmation	Submit all *Corynebacterium diphtheriae* isolates to a reference laboratory for confirmation and toxin determination using phenotypic (Elek test) and genotypic (PCR) methods.
5. Surveillance and reporting	Encourage routine submission of *Corynebacterium diphtheriae* isolates to central reference laboratories for surveillance. If this is not feasible, isolates should be cryo-stored for future studies and research.

MALDI-TOF MS, Matrix-Assisted Laser Desorption/Ionization Time-of-Flight Mass Spectrometry; API, Analytical Profile Index; PCR, polymerase chain reaction.

## Conclusion

Routine species-level identification of *C. diphtheriae* should be prioritised in laboratories, moving beyond classification of diphtheroids as mere contaminants. Diagnostic workflows should incorporate selective media and advanced identification techniques to enhance detection accuracy. National reference laboratories should expand surveillance efforts to track nontoxigenic *C. diphtheriae* cases, enabling a better understanding of disease burden and informing public health policies. Clinicians must remain aware of *C. diphtheriae* as a potential pathogen in bloodstream infections, endocarditis, and soft tissue infections to ensure timely diagnosis and appropriate management. Further research should focus on exploring the molecular epidemiology of nontoxigenic *C. diphtheriae* and standardising detection and treatment protocols.
